# Lung hyperinflation and functional exercise capacity in patients with COPD – a three-year longitudinal study

**DOI:** 10.1186/s12890-018-0747-9

**Published:** 2018-12-06

**Authors:** Linn T. Aalstad, Jon A. Hardie, Birgitte Espehaug, Einar Thorsen, Per S. Bakke, Tomas M. L. Eagan, Bente Frisk

**Affiliations:** 1 Department of Clinical Science, University of Bergen, Bergen, Norway; 2 Centre for Evidence-Based Practice, Western Norway University of Applied Sciences, Bergen, Norway; 3 Department of Occupational Medicine, Haukeland University Hospital, Bergen, Norway; 4 Department of Thoracic Medicine, Haukeland University Hospital, Bergen, Norway; 5 Department of Health and Functioning, Western Norway University of Applied Sciences, Inndalsveien 28, 5063 Bergen, Norway; 6 Department of Physiotherapy, Haukeland University Hospital, Bergen, Norway

**Keywords:** COPD, 6-min walk distance, IC/TLC ratio, Functional exercise capacity, Lung hyperinflation

## Abstract

**Background:**

Lung hyperinflation contributes to dyspnea, morbidity and mortality in chronic obstructive pulmonary disease (COPD). The inspiratory-to-total lung capacity (IC/TLC) ratio is a measure of lung hyperinflation and is associated with exercise intolerance. However, knowledge of its effect on longitudinal change in the 6-min walk distance (6MWD) in patients with COPD is scarce. We aimed to study whether the IC/TLC ratio predicts longitudinal change in 6MWD in patients with COPD.

**Methods:**

This prospective cohort study included 389 patients aged 40–75 years with clinically stable COPD in Global Initiative for Chronic Obstructive Lung Disease stages II-IV. The 6MWD was measured at baseline, and after one and 3 years. We performed generalized estimating equation regression analyses to examine predictors for longitudinal change in 6MWD. Predictors at baseline were: IC/TLC ratio, age, gender, pack years, fat mass index (FMI), fat-free mass index (FFMI), number of exacerbations within 12 months prior to inclusion, Charlson index for comorbidities, forced vital capacity (FVC), forced expiratory volume in 1 s (FEV_1_), and light and hard self-reported physical activity.

**Results:**

Reduced IC/TLC ratio (*p* < 0.001) was a statistically significant predictor for decline in 6MWD. With a 0.1-unit decrease in baseline IC/TLC ratio, the annual decline in 6MWD was 12.7 m (*p* < 0.001). Study participants with an IC/TLC ratio in the upper quartiles maintained their 6MWD from baseline to year 3, while it was significantly reduced for the patients with an IC/TLC ratio in the lower quartiles. Absence of light and hard physical activity, increased age and FMI, decreased FEV_1_ and FVC, more frequent exacerbations and higher Charlson comorbidity index were also predictors for lower 6MWD at any given time, but did not predict higher rate of decline over the timespan of the study.

**Conclusion:**

Our findings demonstrated that patients with less lung hyperinflation at baseline maintained their functional exercise capacity during the follow-up period, and that it was significantly reduced for patients with increased lung hyperinflation.

## Background

Chronic obstructive pulmonary disease (COPD) is a major cause of morbidity and mortality, and is predicted to become the third leading cause of death worldwide in 2030 [1]. Expiratory flow limitation is generally regarded as the hallmark feature of COPD [2], but lung hyperinflation is also a manifestation of COPD with major clinical consequences [3].

Lung hyperinflation is clinically relevant in COPD, mostly because of its contribution to dyspnea [4] and morbidity associated with the disease [5]. Hyperinflation has also shown to be related to exercise limitation [4], which is a significant component of quality of life [6]. The inspiratory capacity (IC) and inspiratory-to-total lung capacity (IC/TLC) ratio are indirect measures of lung hyperinflation reflecting the end-expiratory lung volume [5, 7]. IC and IC/TLC ratio progressively declines as COPD advances [7, 8], mirroring the rise in lung hyperinflation [9].

The 6-min walk test (6MWT) is widely used in the assessment of functional exercise capacity in patients with COPD [10]. The 6-min walk distance (6MWD) gives valuable information that can be used in the clinical staging of COPD patients, as it correlates well with dyspnea, lung hyperinflation and airway obstruction [11]. In addition, longitudinal changes in 6MWD have demonstrated to be important predictors of mortality [12–14]. However, there is limited knowledge about the effect of IC/TLC ratio on longitudinal changes in 6MWD in patients with COPD.

To our knowledge, only one previous study has examined the relationship between lung hyperinflation and functional exercise capacity, using IC/TLC ratio and 6MWD in the assessment. Ramon et al. [15] demonstrated that IC/TLC ratio and subjective dyspnea predicted decline in exercise capacity. However, Ramon et al. [15] only included patients recruited during their first hospitalization due to a COPD exacerbation, and may not be representative for COPD patients in general. The observation time was 1.7 years [15].

The aim of the present study was to examine whether lung hyperinflation measured as IC/TLC ratio predicts longitudinal change in 6MWD in patients with COPD. We hypothesized that increased hyperinflation predicts longitudinal decline in functional exercise capacity in patients with COPD.

## Methods

### Study population

The current study was a prospective cohort study that included 389 of a total of 433 patients from the Bergen COPD Cohort Study (BCCS), aged 44–75 years, with an average follow-up time of 3 years (2006–2010). Details of sampling procedures and data collection for the BCCS have been described previously [16]. In brief, all patients had a clinically stable COPD in Global Initiative for Chronic Obstructive Lung Disease (GOLD) stages II-IV [17], a smoking history of ≥10 pack years, a post-bronchodilation forced expiratory volume in 1 s (FEV_1_)/forced vital capacity (FVC) ratio < 0.7 and a post-bronchodilation FEV_1_ < 80% of predicted value according to Norwegian reference values [18]. Exacerbations that required medical treatment and/or hospitalization during the last 4 weeks prior to inclusion led to postponement of inclusion. There were no restrictions to treatment during the study period, and the patients received medications and therapies prescribed by their physician. Active cancer or inflammatory disorders like rheumatoid arthritis, systemic lupus erythematosus or other connective tissue disorders and inflammatory bowel disease in the last 5 years were reasons for exclusion from the study. The patients were recruited through outpatient clinics from several hospitals in Western Norway and from three private specialist practices in Bergen, Norway [16].

### Measurements

#### Six-min walk test

Functional exercise capacity was assessed by the 6MWT with 6MWD as the primary outcome measure. The 6MWT was performed at baseline and after one and 3 years after inclusion in the study. The test was supervised by a trained technician and performed according to the American Thoracic Society (ATS) guidelines [10]. However, the 6MWT was performed only once at each visit without a prior practice test. The 6MWD and use of oxygen during the test were registered. Measurements of heart rate and oxygen saturation, as well as evaluation of dyspnea and fatigue according to the Borg CR10 Scale [19], were done before and immediately after the test.

#### Self-reported physical activity

Level of habitual physical activity was registered by a questionnaire with two questions related to spare time physical activity, one for hard and one for light physical activity. Sweating and breathlessness were used as the delineation between hard and light physical activity. The possible response categories were none, less than 1 h per week, 1–2 h per week and 3 or more hours per week. These questions are validated previously [20, 21], and have been used in a large Norwegian general population study [22].

#### Body composition, spirometry, dyspnea, exacerbations and Charlson index for comorbidities

We measured height and body mass, and the study participants underwent bioelectrical impedance measurements of fat mass and fat-free mass after an overnight fast (Bodystat 1500, Isle of man, England). Fat mass index (FMI) and fat-free mass index (FFMI) were calculated as fat mass and fat-free mass, respectively, divided by the square of height.

The study participants underwent complete lung function tests at baseline, including spirometry and plethysmography, according to the ATS/ERS Standardization of Lung Function Testing [23, 24]. We performed spirometry before and after inhalation of 0.4 mg salbutamol on a Viasys Masterscope (Viasys, Hoechberg, Germany). The FVC and FEV_1_ were taken as the highest values from at least three acceptable and repeatable maneuvers. Lung volumes were obtained by plethysmography and lung hyperinflation was assessed by the IC/TLC ratio. The spirometers were calibrated twice daily with a 3-L calibration syringe.

The modified Medical Research Council (mMRC) dyspnea scale [25] was used to measure symptoms of dyspnea. A physician examined all the patients at baseline and registered number of exacerbations the last 12 months prior to inclusion, comorbidities and smoking habits. The Charlson index for comorbidities was calculated.

#### Statistics

Descriptive statistics were used to characterize the study population (mean, standard deviation and percent). Normal distribution was assessed by histogram, Q-Q-plot and Shapiro Wilks test. We compared the continuous and categorical variables across gender by independent t-tests and chi-square tests, respectively.

We performed generalized estimating equations (GEE) regression analyses [26] to examine IC/TLC ratio as predictor for change in 6MWD. Time was included in the model as a categorical variable with three measurements: baseline, 1 and 3 years, and in addition as a continuous variable to examine yearly change in 6MWD. Age, gender, pack years, FMI, FFMI, more than two exacerbations within 12 months prior to inclusion (yes/no), Charlson index for comorbidity (level 1, 2, 3 and 4), FVC, FEV_1_ and light or hard self-reported physical activity (yes/no, yes = ≥1 h/week) at baseline were included as potential confounders. To account for within-patient correlation we applied an unstructured working correlation structure. GEE regression analysis provides an approach for analyzing correlated measurements without excluding subjects with an incomplete dataset.

We performed bivariate GEE analysis for each predictor variable. In an additional analysis, time was investigated as a continuous variable. Multivariate GEE analyses were performed in three stages, first including all variables as main effects, and secondly, expanding with all possible interaction terms between predictor and time. Based on the latter, the third and final model included all variables and the statistically significant interaction terms. Analyses for multicollinearity between FMI and FFMI, and between IC/TLC ratio, FVC and FEV_1_ did not demonstrate multicollinearity, and therefore all variables were included in the same model.

In addition, we performed multivariate GEE analyses with baseline IC/TLC ratio categorized into quartiles, and the variable was included in the analysis as the interaction term with time. The IC/TLC groups were named IC/TLC 1, 2, 3 and 4, respectively, with IC/TLC 1 representing the lower quartile.

A power analysis for a simple group comparison with three repeated measures [26] informed that 174 patients were needed to detect a difference in mean 6MWD of 30 m [27] as statistically significant at a 5% significance level and a power of 80%. The calculation was based on an assumption of a within-subject correlation coefficient of 0.25 and a SD for 6MWD of 100 m. A within-subject correlation coefficient of 0.5 increased the number of patients to 232.

IBM SPSS Statistics version 24 was used to conduct the analyses. Estimated regression coefficients obtained in the GEE analyses are presented with confidence intervals and *p*-values. Statistical significance level was set at 0.05.

## Results

### Study population

At baseline, 389 patients completed the 6MWT, and 319 (82%) and 264 (68%) completed the 6MWT at 1 year and 3-years, respectively. Of the remaining 125 participants, 104 were disabled and 21 were deceased. The patients who dropped out were older (*p* < 0.001), had lower FEV_1_ (*p* < 0.001), FVC (*p* < 0.001) and IC/TLC ratio (*p* < 0.001) compared to the patients who completed the study, and the 6MWD were 87 m (*p* < 0.001) shorter at baseline. Baseline characteristics of the study population are presented in Table 1. The mean age of the patients was 64 ± 7 years, and 61% were male. Airflow limitation was moderate to very severe with a mean FEV_1_ of 49 ± 14% of predicted value. Measurements of IC/TLC ratio at baseline were available for 371 patients. The patients had a mean IC/TLC ratio of 0.35 ± 0.09 and approximately 15% of the patients had an IC/TLC ratio below the critical threshold of 0.25. The cut points for the quartiles were 0.29 (25th percentile), 0.34 (median) and 0.40 (75th percentile).

**Table 1:** Baseline characteristics of the study population

Predictors	Total	Female	Male	*P*-value
Sex, n (%)	389 (100)	153 (39.3)	236 (60.7)	
Age (years)	63.6 ± 6.8	62.6 ± 6.3	64.2 ± 7.0	0.028
6MWD (m)	423 ± 112	406 ± 104	434 ± 115	0.015
SpO_2_ before 6MWT (%)	94.2 ± 2.7	94.1 ± 2.9	94.3 ± 2.6	0.621
SpO_2_ after 6MWT (%)	91.0 ± 5.7	90.9 ± 6.1	91.0 ± 5.4	0.804
Borg dyspnea score after 6MWT (median)	4.0	4.0	3.0	0.085
mMRC	2.2 ± 2.3	2.3 ± 2.4	2.1 ± 2.2	0.410
BMI (kg/m^2^)	25.4 ± 5.2	24.5 ± 5.7	25.9 ± 4.8	0.016
FMI (kg/m^2^)	8.4 ± 3.3	9.8 ± 3.7	7.4 ± 2.6	< 0.001
FFMI (kg/m^2^)	17.0 ± 3.2	14.7 ± 2.4	18.5 ± 2.8	< 0.001
Smoking status n (%)				0.215
Current	167 (43)	72 (47)	95 (40)	
Former	222 (57)	81 (53)	141 (60)	
Pack years	40.9 ± 22.8	33.5 ± 16.6	45.6 ± 24.9	< 0.001
GOLD category, n %				0.283
II	179 (46)	76 (50)	103 (44)	
III	169 (43)	65 (42)	104 (44)	
IV	41 (11)	12 (8)	29 (12)	
Charlson comorbidity index, n (%)				0.011
1	225 (58)	99 (65)	126 (53)	
2	93 (24)	38 (25)	55 (23)	
3	46 (12)	12 (8)	34 (15)	
4	25 (6)	4 (2)	21 (9)	
Experienced > 2 exacerbations, n (%)	75 (20)	38 (25)	37 (16)	0.036
FEV_1_ (L)	1.5 ± 0.5	1.3 ± 0.4	1.7 ± 0.5	< 0.001
FEV_1_ (% pred.)	48.7 ± 14.0	49.6 ± 13.5	48.1 ± 14.4	0.315
FVC (L)	3.3 ± 0.9	2.7 ± 0.6	3.7 ± 0.8	< 0.001
FVC (% pred.)	85.5 ± 16.6	85.2 ± 17.1	85.6 ± 16.4	0.791
FEV_1_/FVC (%)	45.7 ± 11.1	47.6 ± 11.0	44.5 ± 11.0	0.006
TLC (L)	7.1 ± 1.4	6.1 ± 0.9	7.8 ± 1.3	< 0.001
TLC (% pred.)	115.9 ± 18.0	121.4 ± 17.8	112.3 ± 17.1	< 0.001
IC (L)	2.4 ± 0.7	2.0 ± 0.5	2.7 ± 0.7	< 0.001
IC/TLC ratio	0.35 ± 0.09	0.36 ± 0.08	0.33 ± 0.09	0.002
TLCO	5.2 ± 1.9	4.4 ± 1.6	5.6 ± 2.0	< 0.001
TLCO (% pred.)	59.1 ± 18.8	57.2 ± 18.5	60.3 ± 19.0	0.143
Physical activity ≥1 h/week				
Light physical activity	263 (73.0)	104 (73.7)	159 (72.6)	0.691
Hard physical activity	141 (39.6)	54 (38.9)	88 (40.6)	0.139

Data are presented as mean ± SD, unless otherwise stated. Independent samples T-test for continuous variables and Chi square for categorical variables

*6MWD* 6-min walking distance, *BMI* body mass index, *FMI* fat mass index, *FFMI* fat free mass index, *GOLD* Global Initiative for Chronic Obstructive Lung Disease, *FEV*_*1*_ forced expiratory volume in 1 s, *FVC* forced vital capacity, *IC* inspiratory capacity, *TLC* total lung capacity, *IC/TLC ratio* inspiratory-to-total lung capacity ratio, *TLCO* transfer factor for carbon monoxide

### Longitudinal change in 6MWD

**Fig. 1:**
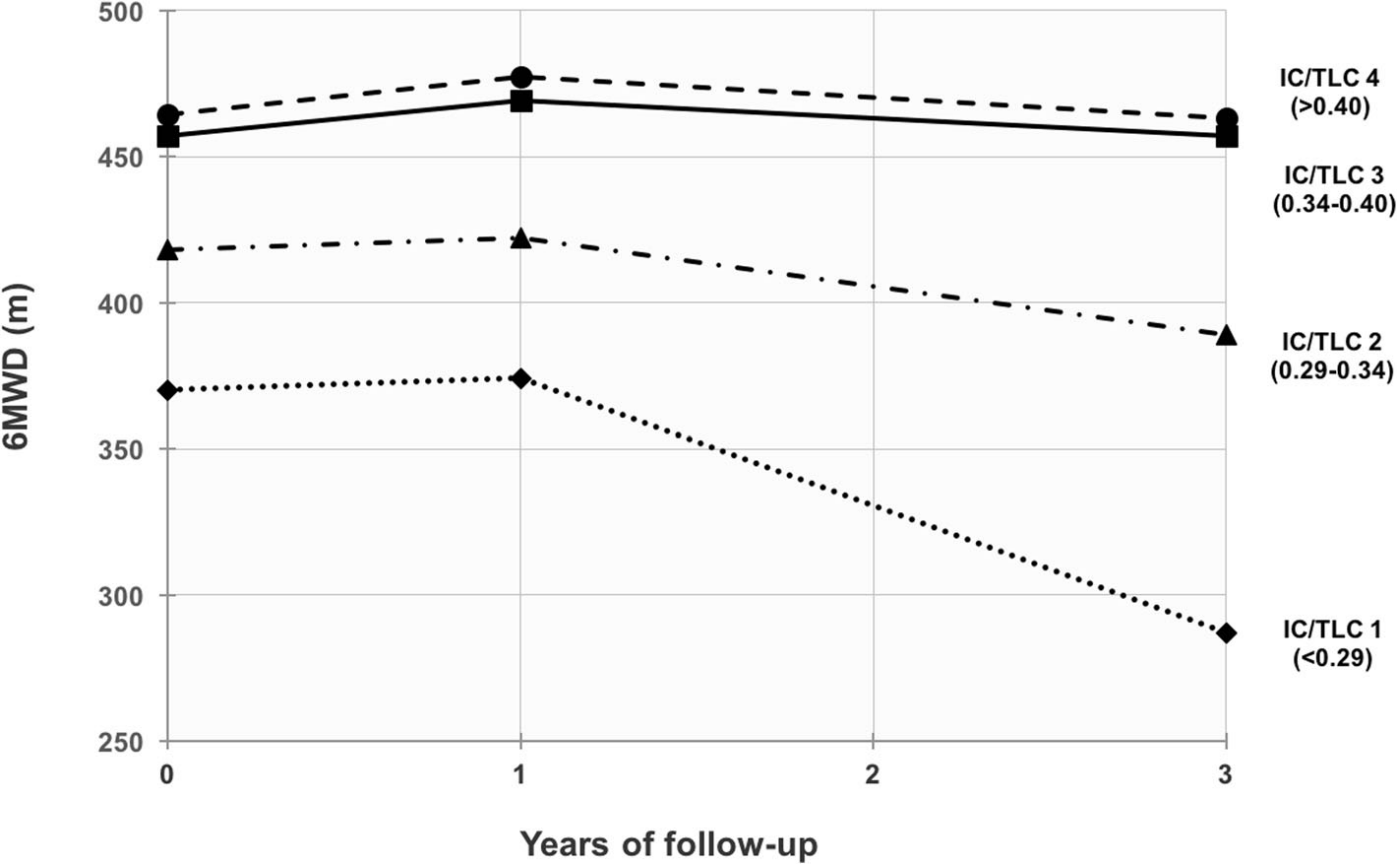
Estimated mean 6-min walk distance (6MWD) by quartiles of inspiratory-to-total lung capacity (IC/TLC) ratio at baseline and during 3 years of follow-up. IC/TLC ratio at baseline was divided into approximate quartiles. IC/TLC ratio and the interaction term with time was included in an unadjusted generalized estimating equations regression analyses

Estimated unadjusted mean (SE) 6MWD at baseline and after 1 and 3 years were 429 (6), 437 (6) and 408 (8) m, respectively. There was a statistically significant annual decrease in 6MWD during the follow-up period of − 10.9 m (95% CI: -15.3 – -6.6, *p* < 0.001). However, mean 6MWD increased slightly from baseline to year 1 (B = 8.1, *p* = 0.05) (Table 2), and was significantly reduced from year 1 to year 3 (B = − 29, *p* < 0.001) indicating a non-linear relationship. This pattern was most evident in patients with an IC/TLC ratio in the lower quartiles (Fig. 1).

**Table 2:** Predictors for mean 6MWD estimated with GEE regression analysis

Predictors	Bivariate		Multivariate^a^		
	Beta	*p*	Beta	95% CI	*p*
Time		< 0.001			< 0.001^b^
1 year vs. baseline	8.1	0.052	6.0	- 1.9 – 13.4	0.134
3 years vs. baseline	- 21.0	< 0.001	- 24.1	- 35.1 – - 13.9	< 0.001
Sex, (male vs. female)	35.1	0.002	- 28.8	- 60.1 – 2.5	0.072
Age (years)	- 5.3	< 0.001	- 1.8	- 3.4 – - 0.3	0.019
FEV_1_ (L)	107.3	< 0.001	47.5	21.7–73.2	< 0.001
FVC (L)	57.1	< 0.001	20.9	5.2–36.7	0.009
IC/TLC ratio (at 0.35)	489.4	< 0.001			< 0.001^c^
At baseline			116.3	−10.0 – 242.7	0.071
At 1 year			175.2	25.1–325.4	0.022
At 3 years			454.2	263.3–645.2	< 0.001
FMI (kg/m^2^)	- 8.2	< 0.001	- 8.3	- 12.2 – - 4.4	< 0.001
FFMI (kg/m^2^)	5.2	0.008	1.3	- 3.6 – 6.3	0.592
Pack years	- 0.2	0.577	- 0.2	- 0.7 – 0.3	0.421
Experienced > 2 exacerbations last year	- 66.4	< 0.001	- 32.1	- 53.5 – - 10.7	0.003
Charlson comorbidity index; score		0.001			0.006^b^
2 vs. 1	- 30.6	0.025	- 12.1	- 33.5 – 9.2	0.266
3 vs. 1	- 56.6	0.003	- 37.4	- 68.7 – - 6.1	0.019
4 vs. 1	- 58.9	0.016	- 54.8	- 91.6 – - 18.0	0.004
Light physical activity ≥1 h/week	69.9	< 0.001	34.2	7.5–60.8	0.012
Hard physical activity ≥1 h/week	66.4	< 0.001	37.0	18.6–55.4	< 0.001

*Beta* estimated regression coefficient, *P p*-value, *CI* confidence interval, *6MWD* 6-min walk distance, *GEE* generalized estimated equation, *FEV*_*1*_ forced expiratory volume in one second, *FVC* forced vital capacity, *IC/TLC ratio* inspiratory-to-total lung capacity ratio, *FMI* fat mass index, *FFMI* fat free mass index

^a^ The model included all predictors and interaction terms for time x IC/TLC ratio centered at mean value (0.35). ^b^ Test for overall effect. ^c^ Test for time as effect modifier (test of interaction)

### Predictors for longitudinal change in 6MWD

A 0.1 unit increase in baseline IC/TLC ratio was associated with an increase in 6MWD both in the bivariate (B = 489 m, *p* < 0.001) and multivariate (B = 229 m, *p* = 0.001) analysis (Table 2). In the final model, we allowed for a time-dependent effect of the IC/TLC ratio, and found that it gained importance as predictor of 6MWD with longer follow-up (Table 2). Among all included factors, only IC/TLC ratio demonstrated a statistically significant time-dependent effect on 6MWD (*p* < 0.001). However, several other baseline factors had a statistically significant impact on 6MWD irrespective of follow-up. We observed that not performing light and hard physical activity, decreasing values of FEV_1_ or FVC, increasing age or FMI, Charlson comorbidity index of 3 or higher, and having more than two exacerbations the last 12 months prior to study inclusion, predicted a statistically significant decrease in 6MWD (Table 2).

## Discussion

The main findings of this study were: 1) Increased lung hyperinflation measured as IC/TLC ratio predicted longitudinal decline in 6MWD, with time as an effect modifier. Absence of light and hard physical activity, increased age and FMI, decreased FEV_1_ and FVC, more frequent exacerbations and higher Charlson comorbidity index were also predictors for lower 6MWD at any given time, but did not predict higher rate of decline over the timespan of the study. 2) Study participants with an IC/TLC ratio in the upper quartiles maintained their 6MWD from baseline to year 3, while it was significantly reduced for study participants with an IC/TLC ratio in the lower quartiles.

### Longitudinal change in 6MWD

The analysis of data with repeated outcome measurements over time is complicated. Baseline predictor variables can be associated to the outcome at any given time point without actually predicting the time-related change in the outcome. It is the interaction of time and the baseline variable (effect modification) which shows whether there is a prediction of change in the outcome over time. In our study, only IC/TLC ratio interacted significantly with time, and as such, was the only variable to actually predict a change in the 6MWD.

To our knowledge, only four previous studies have investigated risk factors for decline in functional exercise capacity assessed by the 6MWT in patients with COPD [12, 15, 28, 29]. Of these, only one study has examined the relationship between lung hyperinflation and functional exercise capacity. Ramon et al. [15] conducted a prospective cohort study with 342 patients with a clinically stable COPD, and a mean follow-up period of 1.7 years. The study demonstrated that IC/TLC ratio and dyspnea score predicted decline in functional exercise capacity measured by 6MWD.

We confirmed the findings from Ramon et al. by demonstrating that IC/TLC ratio predicted longitudinal change in functional exercise capacity. However, patients in the study by Ramon et al. [15] were recruited during their first hospitalization due to a COPD exacerbation, and performed two 6MWTs, the first at least 3 months after discharge from hospital and the second 18–24 months after the first test. The period of follow-up was relatively short. Our study included a wide spectrum of patients with clinically stable COPD in GOLD stages II-IV that performed the 6MWT three times during a follow-up period of 3 years. We also demonstrated, irrespective of follow-up, associations between a decrease in 6MWD and a decrease in FEV_1_ and FVC, increased age, FMI, comorbidity and number of exacerbations and absence of light and hard physical activity.

Previous studies have demonstrated that airflow limitation (lower FEV_1_) and older age were risk factors for longitudinal decline in functional exercise capacity [12, 28, 29]. In addition, Spruit et al. [29] found that lower body mass index (BMI) was a predictor for longitudinal change in functional exercise capacity. Our group has previously demonstrated in the same cohort as in this study, that lower levels of habitual hard physical activity is associated with an increased deterioration in functional exercise capacity [28]. In consistency with previous research [12, 28, 29], we demonstrated in this study that lower FEV_1_ and older age were associated with a lower 6MWD. A lower FEV_1_ is related to reduced maximal expiratory flow rates and impaired ventilatory capacity, being a limiting factor for functional exercise capacity.

Lung hyperinflation predisposes for a mechanical constraint on expansion of the tidal volume as the need for ventilation increases during exercise, resulting in exercise intolerance [9, 30]. Performing light and hard habitual physical activity demonstrated a positive association with 6MWD. However, these results were not significant when analyzing for time as an effect modifier. Physical activity improves exercise capacity and the function of the skeletal and muscle system, but it does not improve the bronchial obstruction in patients with COPD [31].

We did not examine whether BMI was a predictor for change in 6MWD, but included FMI in the analysis, which was a statistically significant predictor at 1 and 3 years. A novel finding from our study is that a higher frequency of COPD exacerbations and a higher Charlson comorbidity index were predictors for a lower 6MWD. As previously demonstrated by Park et al. [8], progression of lung hyperinflation is associated with more frequent exacerbations.

Studies have demonstrated that IC/TLC ratio declines over time in patients with COPD [7, 32], including a recent study conducted by Park et al. [8] that demonstrated a decline in IC/TLC ratio over time at a mean rate of 0.70% per year. As such, one expects that the patients experience a progression of lung hyperinflation during the study period, associated with a decline in IC/TLC ratio [8]. Patients in the lower quartiles of IC/TLC ratio experienced a higher decrease in 6MWD during the follow-up period compared to participants with an IC/TLC ratio in the higher quartiles, who maintained their 6MWD.

Different variables show up as significant predictors in different studies. Whether it is FEV_1_ or IC/TLC ratio, or markers of clinical severity or body composition, they all reflect status of the lung mechanics and clinical condition, which are the two most important factors describing the severity of the disease.

### Functional exercise capacity

Functional exercise capacity is determined by central and peripheral factors, and the mechanism is complex [33]. In patients with COPD, exercise capacity is mainly limited by peripheral muscle fatigue, impaired ventilatory mechanics and gas exchange [33, 34]. With an increased lung hyperinflation, the patients experiences increased work of breathing. The respiratory muscles are placed at mechanical disadvantage due to adaptation of the diaphragm to chronic overload of the respiratory muscles [28, 35, 36]. This results in impaired inspiratory muscle strength [37] and endurance [38], leading to dyspnea [39, 40] and reduced exercise capacity [41]. Breathing becomes more energy demanding and physical activity becomes increasingly uncomfortable, resulting in a decreased level of habitual physical activity, and secondly, a deterioration in functional exercise capacity.

Our results demonstrates that performing habitual physical activity at baseline was associated with 6MWD in a positive manner.

### Lung hyperinflation

IC can be measured by spirometry, and lung hyperinflation is therefore an easy way to evaluate the patient’s disease severity and risk for functional exercise capacity deterioration. Recent studies have demonstrated that IC can be within the normal range in patients with mild airway obstruction in GOLD stage I, although residual volume (RV) and functional residual capacity (FRC) can be increased [8, 42], pointing towards early hyperinflation. To be able to detect early hyperinflation, we chose to analyze IC/TLC ratio, instead of IC alone. We could also have used the RV/TLC ratio as an explanatory variable, which is another widely used index for lung hyperinflation. In order to compare our findings with those of Ramon et al. [15], we chose to use IC/TLC ratio. We performed additional analyses with RV/TLC instead of IC/TLC ratio, yielding approximately the same results.

### Study strengths and limitations

The current study was a large cohort consisting of patients with clinically stable COPD in GOLD stages II-IV. The large sample size, wide spectrum of disease severity and quite even distribution among gender are clear strengths of our study. This prospective study is also one of few to consider the relationship between longitudinal change in 6MWD and IC/TLC ratio.

The 6MWT with 6MWD as main outcome is a widely used measure of functional exercise capacity in COPD, but the cardiopulmonary exercise test (CPET) performed on cycle ergometer or treadmill, is considered as the gold standard for evaluating causes of exercise intolerance in patients with COPD [43]. Peak oxygen uptake (VO_2peak_) is the main outcome of CPET. The association between 6MWD and VO_2peak_ has shown to be moderate to strong [44, 45] and the 6MWD a reliable measure of walking capacity [46, 47].

In healthy persons and patients with mild COPD, the 6MWT often shows a ceiling effect where it is the maximal walking speed rather than the ventilatory capacity that limits the 6MWD [28]. For some of our patients, the 6MWD was not different from the normal population. A longitudinal decline in 6MWD has been demonstrated in previous studies [12, 29], but this finding was only evident in patients with severe airflow obstruction. Even though there was a decline in 6MWD for patients in GOLD stages III and IV, the mean annual decline was less than 30 m that is considered the minimal clinically significant change [27].

The patients were evaluated three times during the follow-up period of 3 years, performing one 6MWT at each visit. Since the test-battery in the BCCS was comprehensive and demanding for the patients, only one 6MWT was performed at each visit rather than two, which is recommended by the ATS guidelines. By doing two tests, any learning effect is reduced. A decline in 6MWD could therefore have been concealed by the learning effect, and could explain our finding of no change in 6MWD after 1 year. However, it is more unlikely that this effect influenced the results after 3 years.

This study was a subsample of the BCCS, in which patients with inflammatory diseases were excluded. However, patients with chronic diseases like heart failure were included in the study. A possible limitation of our study is the possible effect that such comorbid disease could influence longitudinal change in 6MWD. Regardless, our findings are likely generalizable because comorbid diseases are common among patients with COPD and our study population is assumedly representative for common COPD patients. This is also accounted for by including Charlson comorbidity index in the analyses.

Patients were free to receive medication and therapy, such as pulmonary rehabilitation, during the study. This may have influenced the longitudinal change in 6MWD. Frisk et al. [28] reported that patients participating in a pulmonary rehabilitation program during the study period reported a higher level of habitual physical activity at 3 years follow-up. However, hard physical activity and FEV_1_ remained as significant predictors for change in 6MWD when separate analyses were done for patients who did participate in pulmonary rehabilitation and those who did not [28].

Our study had a dropout rate of 32% from baseline to year 3. Most patients were lost to follow-up because of increased disease severity or death. Our dropout rate is comparable to the dropout rate of 31% in the study by Spruit et al. [29], which also had a study period of 3 years. The study by Casanova et al. [12] had a dropout rate of 34% during a study period of 5 years. In studies with COPD patients, an increasing dropout rate is hard to avoid due to progression of the disease during longitudinal studies.

## Conclusion

Baseline IC/TLC ratio was a statistically significant predictor for longitudinal change in 6MWD over a period of 3 years. Patients with less lung hyperinflation at baseline maintained their functional exercise capacity in the follow-up period, while it declined significantly in those with increased hyperinflation.

### Clinical implications

Decline in functional exercise capacity can to a certain degree be prevented by therapeutic interventions such as pulmonary rehabilitation [48]. It is useful to be able to identify patients that are at particular risk for such deterioration, in order to modify the course of disease.

## Abbreviations

6MWD: 6-min walk distance
6MWT: 6-min walk test
ATS: American Thoracic Society
BCCS: Bergen COPD Cohort Study
BMI: Body mass index
COPD: Chronic obstructive pulmonary disease
CPET: Cardiopulmonary exercise test
FEV_1_: Forced expiratory volume in 1 s
FFMI: Fat-free mass index
FMI: Fat mass index
FRC: Functional residual capacity
FVC: Forced vital capacity
GEE: Generalized estimating equations
GOLD: Global Initiative for Chronic obstructive lung disease
IC: Inspiratory capacity
IC/TLC: Inspiratory-to-total lung capacity
mMRC: Modified Medical Research Council
RV: Residual volume
VO_2peak_: Peak oxygen uptake
